# Depression and Obesity: Integrating the Role of Stress, Neuroendocrine Dysfunction and Inflammatory Pathways

**DOI:** 10.3389/fendo.2018.00431

**Published:** 2018-07-31

**Authors:** Silvia R. S. Ouakinin, David P. Barreira, Carlos J. Gois

**Affiliations:** ^1^Faculdade de Medicina, Clínica Universitária de Psiquiatria e Psicologia Médica, Universidade de Lisboa, Lisbon, Portugal; ^2^Serviço de Gastrenterologia e Hepatologia, Centro Hospitalar Lisboa Norte-Hospital de Santa Maria, Lisbon, Portugal

**Keywords:** depression, obesity, stress, inflammation, neuroendocrine dysfunction

## Abstract

Literature on depression and obesity describes the relevance of the hypothalamic pituitary adrenal axis dysfunction, sympathetic nervous system (SNS) activation, and inflammatory processes as well as the interaction of genetic and environmental factors. Recent investigation in obesity highlights the involvement of several regulation systems, particularly in white adipose tissue. The hypothalamic pituitary adrenal axis, gonadal, growth hormone, leptin, sympathetic nervous system and adrenergic, dopaminergic, and serotoninergic central pathways, all seem interconnected and involved in obesity. From another perspective, the role of psychosocial chronic stressors, determining poor mental and physical health, is well documented. Empirical data can support biologically conceivable theories describing how perceptions of the external social environment are transduced into cellular inflammation and depression. Although in neurobiological models of depression, stress responses are associated with neuroendocrine and neuro-inflammatory processes, concerning similar pathways to those described in obesity, an integrating model is still lacking. The aim of this mini-review is to offer a reflexion on the interplay between the neuroendocrine dysfunctions related to chronic stress and the nature of the shared biologic mechanisms in the pathophysiology of both clinical entities, depression and obesity. We highlight dysfunctional answers of mind body systems that are usually activated to promote regulation and adaptation. Stress response, as a mediator between different level phenomena, may undertake the role of a plausible link between psychological and biological determinants of disease. Depression and obesity are major public health issues, urging for new insights and novel interventions and this discussion points to the need of a more in-depth approach.

## Introduction

Depression is diagnosed to almost 4% of the world population, with 16.6% lifetime prevalence rate, having a major impact on social and public health (1, 2).

In neurobiological models of depression, stress responses, although dependent on individual's predisposition, are associated with inflammatory mechanisms promoting the activation of serotoninergic pathways and reducing serotonin availability. The neuroendocrine dysregulation, the immune response, and the neuro-inflammatory processes also determines changes in monoaminergic systems (serotonin and norepinephrine) classically associated with the etiology of depression and different symptom profiles (3–5).

Recent research aims to describe the common pathways to clarity different features presented in literature, assuming the relevance of the hypothalamic–pituitary–adrenal axis (HPA) activation, with the increase in glucocorticoids production, and Sympathetic Nervous System (SNS) activation (6, 7). New paradigms emerge, such as the immunologic, neuroendocrine, and inflammatory ones, through the investigation of pro-inflammatory cytokines, systemic inflammation in metabolic syndrome, the role of other neuropeptides and receptors as well as the interaction of genetic and environmental factors.

Obesity should not be considered just a disease; from an evolutionary point of view it is perhaps better understood as a product of genetic selection promoting thrifty phenotype individuals, actually living in abundance. In fact, as Bjorntorp stated, the recent obesity epidemic might be due to ancient genetic evolutionary changes becoming apparent in a society of prosperity and longer life expectancy. Also, obesity comorbidities may result more from increasingly stressful environmental factors, rather than from a normal genetic mechanism selected to fight energy deprivation and to promote survival (8). Searching for the reasons of obesity will imply looking for the complex relationships between environment and genes, pre-programmed biological determinants and the external context acting upon them as a vulnerability factor and influenced by individual aspects such as cognition and behavior. New issues arise from the obesity epidemic in developed societies, namely the need to reformulate our clinical approach, through a multidisciplinary and preventive perspective.

The aim of this mini-review is to offer a reflexion on the interplay between the neuroendocrine dysfunctions related to chronic stress and the nature of the common biologic mechanisms in the pathophysiology of both clinical entities, depression and obesity.

## The stress response and the neuroendocrine dysfunction

The perception and evaluation of the stressful nature of an event, relies on behavioral and genetic factors, as well as on the previous experiences and individual resources (9, 10).

Acute and chronic stressors are diverse in nature and consequences. Several studies showed mixed findings regarding the nature of stressors, their temporal dimension and the unpredictability of events (11). Psychosocial stressors with a chronic dimension are well documented as determinants of poor mental and physical health, leading to significant burden to health systems, mortality, morbidity and general wellbeing, predominantly in western societies (12–14).

Chronic stressors create a sustained physiological arousal which allows a set of biological and behavioral adaptive responses. However, the evaluation processes and therefore the impact of a stressor on the organism, depends upon cognitive and emotional factors, which modulates the individual experience of a threatening event (10, 11). Stress vulnerability and its role in several diseases is determined by characteristics such as personality (temperament; character) cognitive resources, emotion regulation, coping strategies, and contextual factors such as social support (13, 15).

Chronic psychological or biological stressors, threatening the homeostatic balance, determine an overload state which promotes stability through change—allostasis. The allostatic load represents the cluster of processes allowing an organized and maintained adaptive response from the stressed organism. In this context, the organism exposure to the response mediators (neuroendocrine or immunological ones) can be distressing and disease promoting (16).

The biological adaptive responses are mediated by regulatory mechanisms, globally known as the stress system, which includes Central Nervous System (CNS) coordination of the HPA axis and Autonomous Nervous System (ANS) which are known to be involved in metabolic syndrome, obesity, and depression.

While in normal circumstances the HPA activation suppresses pro-inflammatory and antiviral immune response, in threatening conditions, when exposure to actual or perceived danger is maintained, the HPA axis promotes an increase in inflammatory response. This process, referred as glucocorticoid resistance or glucocorticoid insensitivity, follows the immune cells' loss of sensitivity to the anti-inflammatory effects of glucocorticoids in order to compensate for their persistent secretion. The details for glucocorticoid resistance are not completely understood, but perhaps its purpose represents an adaptive process, as elevations in pro-inflammatory cytokines accelerate wound healing and limit infections, having a protective role (17, 18).

The HPA axis interact with the ANS, contributing to allostasis. Corticotropin-releasing factor activation increases norepinephrine levels, regulating pro-inflammatory cytokine production. Once released, norepinephrine modulates immune response gene transcription mostly via stimulation of β-adrenergic receptors (19). Sympathetic activation determines an increase in blood pressure and heart rate, and also inhibits the parasympathetic branch of the ANS which modulates immune responses through both the efferent and afferent fibers of the vagus nerve, enabling it to prevent excessive inflammation. Slavish and Irwin (18) state that these data can support an empirical basis for biologically conceivable theories describing how perceptions of the external social environment are transduced into cellular inflammation and depression, proposing their integration into a “social signal transduction theory of depression.” The authors proposed that social threatening situations are represented in brain regions which process experiences of negative affect and rejection-related distress. The connections to lower level brain regions, including the hypothalamus and brainstem regions do not directly regulate inflammatory activity, but they influence systemic inflammation by modulating the activity of the HPA axis and the SNS. While cortisol suppresses inflammatory activity, epinephrine, and norepinephrine promotes inflammation by interacting with immune systems cells through specific receptors.

## Stress, depression, and inflammation

It is now evident that major life stressors can result in homeostatic imbalance and abnormal immune responses, increasing inflammatory activity, and resulting in mental disorders namely depression (20).

Findings linking inflammation and depression are well established and described by several authors since the 1990's (21, 22). This evidence comes from the high levels of inflammatory markers in patients with depression even in the absence of other pathologies, the co-occurrence of depression and inflammatory diseases and the increased risk of depression in patients treated with cytokines (23).

The results of a meta-analysis of 24 studies measuring cytokines in depressed patients, found that individuals with Major Depression had significantly higher concentrations of Tumoral Necrosis Factor alpha (TNF-α) and Interleukin 6 (IL-6) compared to controls (24). Increased peripheral inflammatory markers were found among antidepressant non-responders more often than those who responded to treatment. (3, 25, 26). Higher levels of pro-inflammatory cytokines, Interleukin−1 beta (IL-1β), IL-6 and TNF-α were found in the blood or in the brain of these patients (21, 27, 28). Depression is also accompanied by an increase in acute phase proteins such as haptoglobin, α1-antitrypsin, ceruloplasmin, and C-reactive protein (27, 29).

Cytokines are potent modulators of behavior and mood, and play a central role in the immune system and inflammatory response (21). There are several examples of this in animal models research (30) as well as in cancer or Hepatitis C patients treated with interferon-α (IFN) which is associated with a high incidence of depression (31). Over the last years, research attempting to elucidate the mechanisms involved in the serious collateral effects associated to this agent, namely cognitive disorders and depression has increased. IFN induces changes in the endocrine function (hypothalamic-pituitary-adrenal axis) and in neurotransmission activity (especially serotonin and dopamine) (32–34).

The mechanism by which depression is induced by IFN is still being researched and it is, very likely, multifactorial (34, 35). In agreeance with the literature about the relation between inflammatory cytokine and the serotonin pathways (5-HT), evidence shows that IFN can affect the expression of serotonergic 1A receptors (5-HT1A) (33, 36), which is consistent with what is observed in depressed individuals (37). IFN also reduces the levels of peripheral tryptophan, an effect that is correlated to depression (38).

These data suggest that different physiopathological pathways may be connected to the development of specific symptomatic dimensions, including mood/cognitive symptoms *versus* neurovegetative symptoms, in the context of the cytokine activation system. In addition, Schmidt et al. (39) pointed to the need of a biomarker panel for depression which can perhaps allow for the recognition of a biological signature of major depression subtypes.

## Obesity and depression—NEURO-inflammatory and endocrine pathways

Recent investigation suggests the involvement of several regulatory systems in obesity, particularly in white adipose tissue (WAT). HPA axis, gonadal, growth hormone, leptin, SNS, and adrenergic, dopaminergic, and serotoninergic central pathways, all seem interconnected and involved with obesity. Genetic factors are also relevant, and recent data highlights the glucocorticoids, dopamine or leptin receptors' role (40, 41).

In animal models, stress increases the release of neuropeptide Y, which promotes the growth and differentiation of adipocytes and angiogenesis in the presence of high fat and sugar diet. Prolonged activation of neuropeptide Y and its receptor system (NPY–NPY2R) in adipocytes and endothelial cells is associated to the increase of adipose tissue and metabolic syndrome (42). Adipose tissue, producing, and releasing a variety of hormones and peptides is understood as an endocrine organ, integrating the communication network between peripheral organs and the CNS (43).

Persistent inflammation associated to the increase in adipose tissue can be due to pro-inflammatory cytokines such as TNF-α and IL6 produced by the adipose tissue itself, explaining the neuroendocrine activation and the lipids or glucose metabolism changes observed in obesity (44). The hyperactivation of HPA axis can provoke obesity according to homeostatic and non-homeostatic pathways. The first includes corticotrophin releasing hormone (CRH) suppression, leptin resistance, and increased NPY release. Non-homeostatic pathways include food associated reward and pleasure (dopaminergic and opioidergic pathways) inducing a shift to a hypercaloric diet. In societies presenting high levels of stress and easy available high caloric food, activation of HPA axis might be an important contributing factor to the obesity epidemic (45).

The WAT is a mosaic of adipocytes, nervous tissue, immune cells, connective tissue matrix, and stromovascular cells (46).

Adipokines are proteins specifically secreted from the WAT adipocytes with a local and systemic action. There are pro-inflammatory and anti-inflammatory adipokines. The main examples are leptin, resistin, and adiponectin (47). Leptin is a pro-inflammatory adipokine that regulates dietary intake trough leptin receptors located in the hypothalamus. It promotes the sensation of satisfaction and also increases energy expenditure. Resistin, a pro-inflammatory adipokine increases the secretion of IL-1, IL-6, and TNF-α from macrophages and simultaneously rises its level by the action of these same cytokines (47, 48). Adiponectin, with a predominantly anti-inflammatory role, is reduced in obese persons, inhibiting Th1 responses, polarizating pro-inflamatory M1 macrophages to the anti-inflammatory M2 type, IL-6 and TNF-α production and an increase in cytokine IL-10 secretion (47, 48).

This shift from a physiological toward a dysfunctional expression of adipokines follows obesity where a hypertrophy of the adipose tissue induces hypoxia and an inflammatory response. An infiltration of macrophages changes the profile of the adipose tissue to a pro-inflammatory one. There is a dampening of the expression of adiponectin, whilst increasing the secretion of leptin, IL-6, TNF-α, and PAI-1 (49).

Beyond its peripheral role, systemic cytokines such as IL-6, TNF-α derived from adipose tissue also have access to the CNS leading to activation of microglia which turns the inflammation a central one (50).

The blood brain barrier (BBB) is not a passive structure and reacts to stimuli, changing its permeability and secreting inflammatory mediators to both the circulation and CNS leading to neuroinflammation (50). The pro-inflammatory cytokines can activate indoleamine 2,3-dioxygenase (IDO) and induce neuroinflammation through the synthesis of neurotoxic tryptophan catabolites (TRYCATS), including kynurenine, 3-hydroxykynurenine and quinolinic acid. It has been suggested that depression is associated with these neurotoxic products' consequences to the brain, in addition to the depletion of serotonin, resulting from the increased tryptophan catabolism linked to the IDO functioning (51).

Obesity also initiates an increased immune response against lipopolysaccharides (LPS) of different commensal gram negative bacteria. This immune response against LPS suggests that bacterial translocation to mesenteric lymph nodes or into the systemic circulation might take place, when a “leaky gut” develops with obesity, with increased permeability of the gut wall and a change of the usual intestinal microbiota (52). This immune response will raise the level of circulating systemic cytokines (49). The mucosal intestinal status is sent to the brain via vagal afferent neurons, another communication path between the CNS and the periphery, leading to brain adaptation to inflammation (53).

## Integrating the models

**Figure 1:**
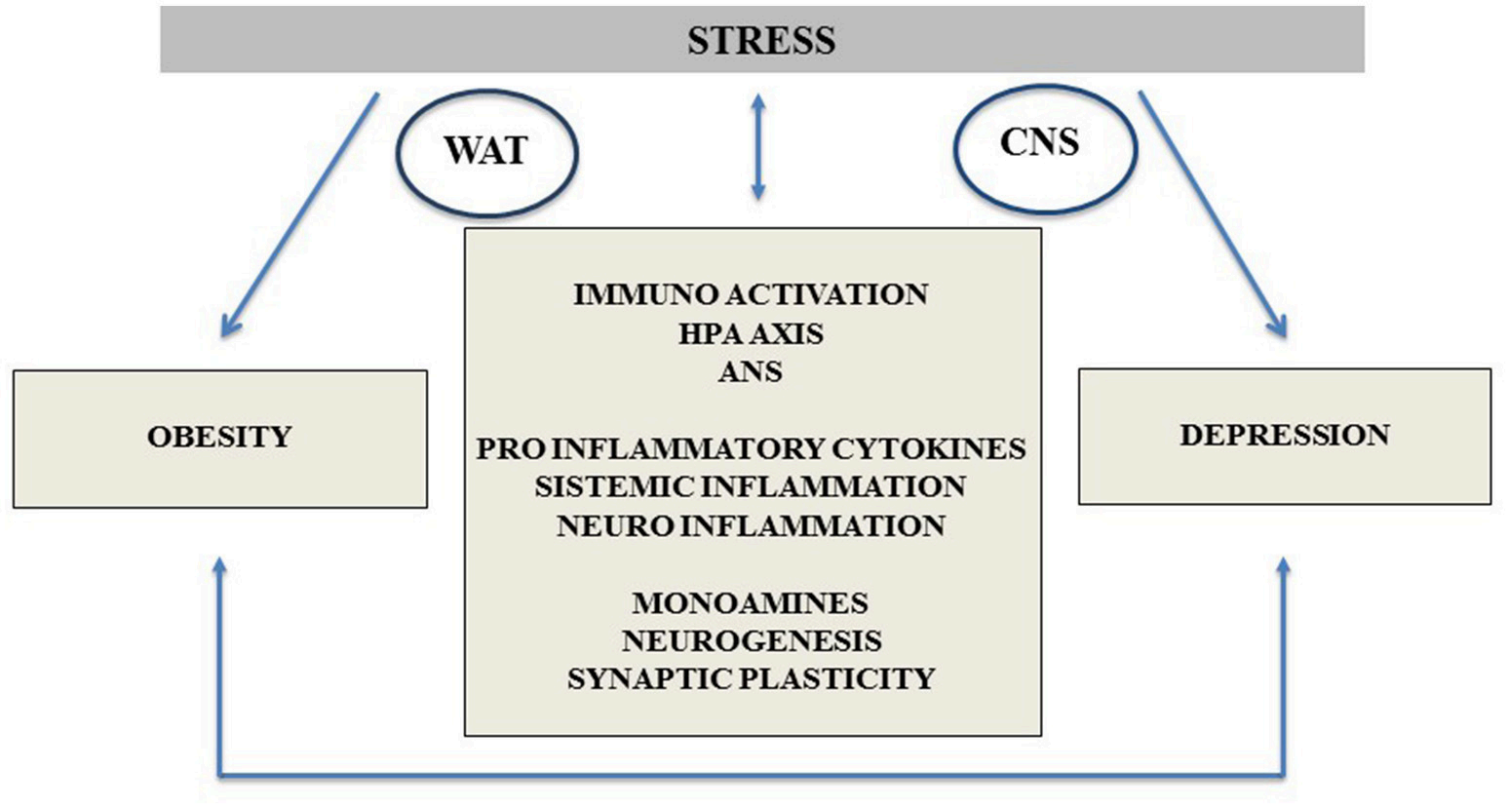
Depression and obesity common neurobiological pathways, highlighting the stress response mechanisms and its impact on the comorbidity between both entities. CNS, central nervous system; WAT, white adipose tissue; HPA Axis, hypothalamic pituitary adrenal Axis; ANS, autonomous nervous system.

From the previous review we can conclude that the relationship between chronic stress, depression, obesity, and its autonomic and neuroendocrine mediation is well documented, as schematically presented in Figure 1.

However, depression and obesity are heterogeneous disorders/conditions and it seems that current neurobiological models lack sufficient power or specificity to explain the diversity of the clinical presentations and interactions.

In recent research the stress-diathesis model of depression was tested, pointing to a significant interaction between polygenic risk scores and personal life events, contributing to a higher risk of depression (54). Clusters of vulnerability, or different diathesis, emphasize the role of personality and affective traits, such as high anxiety trait, as a key vulnerability phenotype to stressful events in the etiology of depression (55). In fact, a previous affective style can amplify the impact of major or minor repetitive stressors, imposing an overload to stress regulatory systems.

Stress can cause depression and other comorbidities through neurobiological pathways leading to neuroinflammation, but also through behavioral ones. In what concerns these later pathways, the relevance of emotional eating in overweight and obesity, triggered by stressful life events and negative affect, seems to lead to an interesting approach. Human studies on the effects of stress in eating behavior, specifically emotional eating, reveal contradictory results. However, they point to individual differences in stress reactivity and to an interaction between cognitive (attentional bias, escape from threatening stimuli), affective (negative affect, emotion regulation, and coping strategies) and a biologic susceptibility (tryptophan and serotonin levels as well as genetic influences on serotonin transporters) (56). Recent research demonstrates that, in women, emotional eating as a psychological eating style, can act as mediator between depression and weight gain (57).

A positive association was found between depression and obesity in the general population, although more marked among women (58). Another study found that individuals with increased psychological distress or depression and a greater polygenic load for obesity were more likely to become obese (59). A profile of moderate depressive symptoms was differentially more associated with obesity when compared with an acute profile of depressive symptoms; if inflammation was controlled for, this link was attenuated. Distinct symptoms profiles may point toward different pathways to increase risk of obesity (60).

A putative role for leptin, adiponectin, and resistin in the pathophysiology of neuropsychiatric conditions associated with metabolic abnormalities, including major depression has emerged. Nonetheless, there are currently no validated peripheral biomarkers for the diagnosis, treatment selection and response prediction in major depression. Detection of inflammatory adipokines and cytokines, like adiponectin, leptin, and resistin, as well as IL6 and C-reactive protein peripheral levels, might fill this gap (61).

Managing obesity can help reduce the risks of other morbid conditions such as depression by inhibiting inflammatory mechanisms associated with obesity (62). Nevertheless, the link between depression and obesity needs further research. Some studies found conflicting and diverging results with the therapeutic approach of extreme obesity. Both increasing and decreasing depressive symptoms were associated with weight loss (63, 64).

In what concerns antidepressants and the bidirectional link between obesity and depression, a recent review highlight the need to control for several confounders, such as age, gender, hormonal status, profile of symptoms, doses and mechanism of action of different drugs. Although a trend to the association of treatment failure and obesity is showed in several studies, a definitive conclusions is still not possible (65).

Considering the higher risk of depression in patients with severe obesity (66), treatment modalities should be tailored according to the needs imposed by such an association.

From a developmental point of view, genetic dispositions and early life adversity can lead to maladaptive stress responses in adulthood, increasing stress vulnerability and amplifying the impact of negative life events (67, 68), leading to depression and also to a psychoneuroimmune dysregulation, promoting obesity. On the other hand obesity, increasing neuroinflammation, may impair monoaminergic neurotransmission, neurogenesis, and synaptic plasticity, potentiating the risk of depression (30).

The search for biomarkers of major depression, using genomic and transcriptomic methodologies, is a promising avenue for the future. However, studies conducted in the last years, although supporting the inflammatory model and identifying potential markers of depression, does not offer a clear and unequivocal profile (69–71).

In summary, despite different levels of evidence, we argue that comorbidity between obesity and depression relies on the association between neuroendocrine and immunologic regulation in allostatic states, in the context of genetic vulnerabilities and behavioral responses to chronic stress. Translational research, improving our understanding of the underlying mechanisms and their clinical implications may contribute to the development of new treatment and prevention strategies.

## Conclusions

Current research highlights the relevance of neuroendocrine and immunologic disruption in several diseases, and inflammation may represent the common mechanism relating different features of premorbid and pathological states. Stress response as a mediator between different level phenomena assumes the role of a plausible link between psychological and biological determinants of health and disease.

Depression and obesity share alterations in cytokine systemic profiles, activation of inflammatory and immune pathways as well as in neuroinflammation, perpetuating the cycle of central/periphery pathogenic interactions. Medical comorbidities associated to obesity and depression are related to a cluster of risk factors, including the metabolic syndrome, associated to the increased morbidity and mortality caused by diabetes, cardiovascular diseases and some cancers (72), all sharing an inflammatory background. Individual variability may be related to psychosocial variables that can amplify the biologic vulnerability, genetically determined or under developmental constrictions.

In the following decades, depression and obesity are expected to represent major public health issues urging for new insights and integrated interventions, both in pharmacological and psychosocial levels.

## Author contributions

All authors listed have made a substantial, direct and intellectual contribution to the work, and approved it for publication.

### Conflict of interest statement

The authors declare that the research was conducted in the absence of any commercial or financial relationships that could be construed as a potential conflict of interest.
